# Amniotic Mesenchymal Stem Cells Can Enhance Angiogenic Capacity via MMPs *In Vitro* and *In Vivo*

**DOI:** 10.1155/2015/324014

**Published:** 2015-09-27

**Authors:** Fei Jiang, Jie Ma, Yi Liang, Yuming Niu, Ning Chen, Ming Shen

**Affiliations:** ^1^Jiangsu Key Laboratory of Oral Diseases, Nanjing Medical University, No. 140, Han Zhong Road, Nanjing, Jiangsu 210029, China; ^2^Department of Dental Implant, Affiliated Hospital of Stomatology, Nanjing Medical University, No. 140, Han Zhong Road, Nanjing, Jiangsu 210029, China; ^3^Department of Stomatology, Taihe Hospital, Hubei University of Medicine, No. 32, Renmingnan Road, Shiyan, Hubei 442000, China

## Abstract

The aim of this study was to evaluate the angiogenic capacity and proteolytic mechanism of coculture using human amniotic mesenchymal stem cells (hAMSCs) with human umbilical vein endothelial cells (HUVECs)* in vivo* and* in vitro* by comparing to those of coculture using bone marrow mesenchymal stem cells with HUVEC. For the* in vivo* experiment, cells (HUVEC-monoculture, HUVEC-hAMSC coculture, and HUVEC-BMMSC coculture) were seeded in fibrin gels and injected subcutaneously in nude mice. The samples were collected on days 7 and 14 and histologically analyzed by H&E and CD31 staining. CD31-positive staining percentage and vessel-like structure (VLS) density were evaluated as quantitative parameters for angiogenesis. The increases of CD31-positive staining area and VLS density in both HUVEC-hAMSC group and HUVEC-BMMSC group were found between two time points, while obvious decline of those was observed in HUVEC-only group. For the* in vitro* experiment, we utilized the same 3D culture model to investigate the proteolytic mechanism related to capillary formation. Intensive vascular networks formed by HUVECs were associated with hAMSCs or BMMSCs and related to MMP2 and MMP9. In conclusion, hAMSCs shared similar capacity and proteolytic mechanism with BMMSCs on neovascularization.

## 1. Introduction

Bone defects remain a major clinical problem in patients' functional reconstruction and remodeling appearance. Bone tissue engineering and regenerative medicine based on stem cells combined with tissue-engineered scaffolds and cytokines have shown a promising potential in regenerating bone defects [1]. Bone is a vital organism that needs blood for material exchange to maintain normal metabolism. Typically, bone is supplied with an intraosseous vasculature with osteocytes at a distance of maximum 100 *μ*m from an intact capillary [2]. However, only cells at the surface of cell-loaded grafts* in vivo* can obtain sufficient blood and nutrient supply to maintain their metabolism and function within a distance of 100–200 *μ*m to the nearest capillaries while cells in the core of the grafts may die [3, 4]. Thus, angiogenesis is a key factor in regeneration medicine.

Therapeutic angiogenesis has been developed as a possible means to treat ischemic diseases via the delivery of proangiogenic molecules to promote neovascularization and tissue repair [5]. Although some strategies have been established to sustain delivery of proangiogenic factors or genes from biodegradable scaffolds [6, 7] and mimic the process of natural vessel development in some degree [8], vascularization* in vivo* triggered by complex proangiogenic signal network still cannot fully reappear by offering combinations of multiple factors. Cell-based therapies have also been explored to more completely mimic the cascade of signals needed to promote the formation of stable neovasculature [9]. A variety of cell types have been shown to form new capillary networks and/or induce collateral blood vessel development after implantation* in vivo *[10–12] and* in vitro* [13]. Their findings were consistent that codelivery of endothelial cells (ECs) and a secondary mesenchymal cell type (e.g., BMMSCs [14–16], AdSCs [17, 18], NHLFs [19], and SMCs [20]) produces the necessary cues to induce tubular sprouting of ECs and stromal cell differentiation toward a pericytic phenotype [21].

The application of mesenchymal stem cells (MSCs) has drawn considerable research interest in bone tissue engineering and regenerative medicine relies on their characteristics of self-renewal and multidirectional differentiation. It has been established that MSCs could be isolated from several tissues, including bone marrow, peripheral blood, and adipose tissue [22]. Although MSCs obtained from these tissues show promising prospect, their application also shows some limitations, where the procedures required to obtain the above tissues are invasive, the number of MSCs obtained is low, and the potential to proliferate and differentiate diminishes as the donor's age increases [23]. Human term placenta has recently attracted wide attention as a valuable source of stem/progenitor cells. It is routinely discarded postpartum as biological waste and is easy to gain without invasive procedures and its use is free of ethical concerns [24].

It had been reported that amniotic membrane-derived mesenchymal stem cells (AMSCs) have potential of osteogenic, adipogenic, chondrogenic, and myogenic differentiation. In addition, Alviano et al. found AMSCs could differentiate into ECs by exposure to VEGF in angiogenic experiments [25]. AMSCs have the higher angiogenic and chemotactic properties compared to adipose tissue-derived MSCs (AdSCs) [26]. AMSCs implantation also augmented blood perfusion and increased intraneural vascularity [27]. However, regarding their angiogenic potential, hAMSCs had been isolated and induced by endothelial growth medium (EBM-2). Induced hAMSCs changed their some mesenchymal phenotype and showed EC-like behavior, but they did not express the mature EC markers [28]. Thus, these findings may support hAMSCs as stromal cells to enhance the viability, sprouting of ECs and promote vessel formation indirectly. In this study, we established 3D culture system to investigate the enhancement of vessel formation by hAMSC* in vivo* and* in vitro*.

## 2. Materials and Methods

### 2.1. Cell Culture

Bone marrow-derived mesenchymal stem cells (BMSCs, passage 2) were tested by the manufacturer (Cyagen Biosciences Inc., Guangzhou, China) for purity by flow cytometry and for their ability to differentiate into osteogenic, chondrogenic, and adipogenic lineages. Cells are positive for the cell surface markers CD105, CD166, CD29, and CD44 and negative for CD14, CD34, and CD45.

### 2.2. Isolation and Culture of hAMSC

Human term placentas and umbilical cords were harvested from normal pregnancies (range 38 to 41 weeks) after spontaneous delivery or cesarean section with informed consent. Approval of the Ethical Committee of Nanjing Medical University was granted. Isolation of hAMSC was performed following a previously established protocol [22]. Briefly, the decidua parietalis was removed by careful scraping. The amnions were cut into small pieces (2∗2 cm) and then manually separated and washed extensively in phosphate-buffered saline containing 100 U/mL penicillin and 100 *μ*g/mL streptomycin (Beyotime, China). Amnion fragments were incubated for 7 min at 37°C in PBS containing 2.4 U/mL dispase (Roche, Mannheim, Germany). The incubated fragments were transferred to *α*-MEM (GIBCO, USA) supplemented with 10% heat-inactivated fetal bovine serum (FBS; GIBCO, USA) for resting 5–10 min at room temperature. After the resting period, the fragments were digested with 0.75 mg/mL collagenase (Roche) and 20 *μ*g/mL DNAse (Roche) for approximately 3 h at 37°C. Amnion fragments were then removed and mobilized cells were passed through a 100 *μ*m cell strainer (BD Falcon, Bedford, MA) and the cells were collected by centrifugation at 200 ×g for 10 min. The cells were cultured in *α*-MEM and used before passage 10.

### 2.3. Isolation and Culture of HUVEC

Human umbilical vein endothelial cells (HUVECs) were harvested from fresh umbilical cords following a previously established protocol [14]. Fresh umbilical veins were digested with 0.1% (w/v) collagenase type 1A solution at 37°C for 15 min to release endothelial cells from the vessel walls. And then, the vessels incubated with collagenase type 1A were rinsed with ECM (ScienCell, San Diego, USA); HUVECs were collected by centrifugation at 1000 rpm for 5 min and cultured in ECM. HUVECs were used at passage 3.

### 2.4. Flow Cytometry

To evaluate cell-surface marker expression, the samples were analysed on a FACSCalibur cytometer and the resulting data were processed using CellQuest software (BD Biosciences). The cell suspensions were incubated for 20 min at 4°C with fluorescein isothiocyanate- (FITC-) or phycoerythrin- (PE-) conjugated monoclonal antibodies specific for human markers associated with mesenchymal and haematopoietic lineages. The antibodies used were CD29(FITC), CD34(FITC), CD44(FITC), CD45(FITC), CD73(PE), CD90(PE), CD105(PE), and HLA-DR(PE) (all from Miltenyi Biotec, Bergisch Gladbach, Germany).

### 2.5. Tissue-Construct Implantation

Animal procedures were performed in accordance with the guidelines for laboratory animal usage following a protocol approved by the Nanjing Medical University's Committee on Use and Care of Animals.

The course of implantation was performed following a previously established protocol [9]. Male 8-week-old BALB/c-nu mice (Jiangsu Provincial Experimental Animal Base for Medicine and Pharmacy) were used for all experiments. Chloral hydrate was delivered to each mouse via intraperitoneal injection before implant injection. In this study, a 2.5 mg/mL plasminogen-depleted human fibrinogen (Sigma-Aldrich, USA) was made in serum-free ECM and filtered through a 0.22 *μ*m syringe filter. Cell mixtures in a 1 : 1 ratio of EC : MSCs (hAMSCs or BMMSCs) were spun down and resuspended in the previously prepared fibrinogen solution at a final concentration of 10 million cells/mL, totaling 3 × 10^6^ cells per injection sample (300 mL total volume). Immediately before injection, 12 *μ*L of thrombin solution (25 U/mL; Sigma-Aldrich, USA) was added to 300 *μ*L of fibrinogen-cell solution. For control samples, 3 million ECs without any stromal cell type were used. Solutions were immediately injected subcutaneously on the dorsal flank of the mouse, with two implants per animal. Animals were kept stationary for 5 min to allow for implant polymerization and were then placed in fresh cages for recovery. Three replicates of each sample type were completed (HUVEC-hAMSC, HUVEC-BMMSC, and HUVEC-only).

### 2.6. Histology and Immunohistochemistry

For histology and immunohistochemical staining, explants were fixed in formalin overnight and then transferred to a PBS, pH 7.4, solution, all at 4°C. All samples were embedded in paraffin and then sectioned in 5 *μ*m sections and stained with hematoxylin and eosin (H&E) and immunohistochemically (hCD31). HE staining was performed according to a standard protocol from pathology department of Nanjing Medical University. For CD31 staining, paraffin sections were rehydrated in serials of ethanol and antigen was retrieved by heating the slides in sodium citrate buffer (PH 6.0) at 97°C for 15 min. Subsequently, blocking was carried out using 5% BSA, and the primary antibody (rabbit anti-human CD31, Abcam, USA) was diluted 1 : 100 in PBS and incubated at 4°C overnight. Slides were then treated with a peroxidase-conjugated AffiniPure goat anti-rabbit IgG (ZSGB-BIO, China) at 1 : 500 for 1 h at room temperature, followed by counterstaining with hematoxylin. Negative controls using PBS instead of the primary antibody were generated in parallel to ensure that the staining was specific. Finally, the sections were dehydrated and mounted. Stained sections were photographed with a Zeiss Imager Z1 microscope equipped with the AxioCam MRc5 camera using AxioVision 4.8 software (Carl Zeiss Microimaging GmbH, Göttingen, Germany).

### 2.7. Histomorphometrical Evaluation

For* in vivo* samples, histomorphometrical analysis was performed to evaluate the angiogenic capacity of three groups (HUVEC-only, HUVEC-hAMSC, and HUVEC-BMMSC) based on hCD31 staining (*n* = 3 per sample) [29]. In brief, the sections were scored using computer-based image analysis techniques (Leica Qwin Proimage analysis system, Wetzlar, Germany), which recognize human endothelial marker (hCD31, stained as brown) within the collagen gels based on different RGB values from highly magnified (200x) digitalized images. Manual corrections were applied to ensure the precise selection of hCD31 staining positive parts within the region of interest (ROI, i.e., the total scaffold area). For the evaluation, VLS (from human origin) were defined by the presence of hCD31 positive (evidenced by hCD31 positive staining) structures with lumens and VLS density: VLS number/ROI (mm^2^).

### 2.8. 3D Fibrin Vasculogenic Assays

Fibrin gels were prepared using plasminogen-depleted human fibrinogen in serum-free ECGM at a concentration of 2.5 mg/mL and filtered for sterility. HUVEC was labeled GFP by using lentivirus before this assay. Cells mixtures in ratios of 5 : 1 of GFP-HUVECs : MSCs (hAMSCs or BMMSCs) were resuspended in prepared fibrinogen solution [30], totaling 2 × 10^4^ GFP-HUVECs and 0.4 × 10^4^ stromal cells per well of 96-well plate (100 *μ*L total volume per well). For control samples, 2.4 × 10^4^ GFP-HUVECs were resuspended in prepared fibrinogen solution (100 *μ*L total volume per well). 3 *μ*L of thrombin solution (25 U/mL; Sigma-Aldrich) was added to 100 *μ*L of fibrinogen-cell solution to catalyze the fibrinogen gel. Prior to fibrin polymerization, the plates were gently tapped on all four edges to assure that the fibrin gel is equally distributed throughout the well. After 30 minutes of incubation at 37°C, gels were fed with 100 *μ*L ECGM. Medium (ECM, ScienCell, USA) was changed at day 3. For studies involving MMPs inhibitor, GM6001 (Calbiochem, San Diego, USA) was added to fibrin gels at final concentration 10 *μ*M. Each group set up 3 repeats. Fluorescent images were captured using a fluorescence inverted microscope (Leica) at 0 h, 12 h, 24 h, 48 h, 72 h, and 96 h, each repeat in 3 random sights being selected to quantitate EC tube area during the morphogenic process by analysis software Image J.

### 2.9. 3D Cell Cluster Formation Assay

To examine the proteolytic function of MSCs in coculture system, we compared MMPs expression in 3D clusters of 3 groups (HUVEC-only, HUVEC-hAMSC, and HUVEC-BMMSCs)* in vitro* after culturing for three days. Fibrin gels were prepared using plasminogen-depleted human fibrinogen in serum-free *α*-MEM at a concentration of 2.5 mg/mL and filtered for sterility. The cell density of seeding and the volume of fibrin gels were just like* in vivo* experiment. 12 *μ*L of thrombin solution (25 U/mL; Sigma-Aldrich) was added to 300 *μ*L of fibrinogen-cell solution to catalyze the fibrinogen gel. After 30 minutes of incubation at 37°C, the gels were transferred from centrifuge tube to culture dish and fed with 5 mL complete *α*-MEM. For 72 h culture, the cell clusters were collected to measure diameter and extracted total protein. The corresponding control groups with 3D cell clusters were fibrin gels without cells.

### 2.10. Western Blotting

Total protein was extracted from cells using lysis buffer (Beyotime, China). Coomassie Brilliant Blue was used to quantify the protein content. The proteins (10 *μ*g) were resolved using sodium dodecyl sulfate-polyacrylamide gel electrophoresis (SDS-PAGE) with 10% polyacrylamide gels and then transferred to polyvinylidene difluoride (PVDF) membranes (Millipore, Billerica, MA, USA), which were blocked with 5% nonfat milk in phosphate-buffered saline (PBS) containing Tween-20 (PBS-T) for 2 h at room temperature. The blots were then probed with primary antibodies specific for MMP2 (1 : 100, Boster, China), MMP9 (1 : 100, Boster, China), and *β*-actin (1 : 1000; Bioworld, USA) overnight at 4°C, washed twice with PBST, and incubated with horseradish peroxidase-conjugated (HRP) secondary antibodies for 1 h at room temperature. Finally, the protein bands were detected by Immobilon Western Chemiluminescent HRP Substrate (Millipore) and visualized using the ImageQuantLAS 4000 mini imaging system (General Electrics, USA). Three independent trials of each experiment were carried out.

### 2.11. Statistical Analyses

All the quantitative results were obtained from triplicate samples. Data are expressed as mean ± standard deviation. Statistical analysis was carried out by use of two-sample *t*-test for comparing two groups of samples and one-way analysis of variance (ANOVA) for three groups. A value of *P* < 0.05 was considered to be statistically significant.

## 3. Results

### 3.1. Identification of hAMSCs and HUVECs

**Figure 1:**
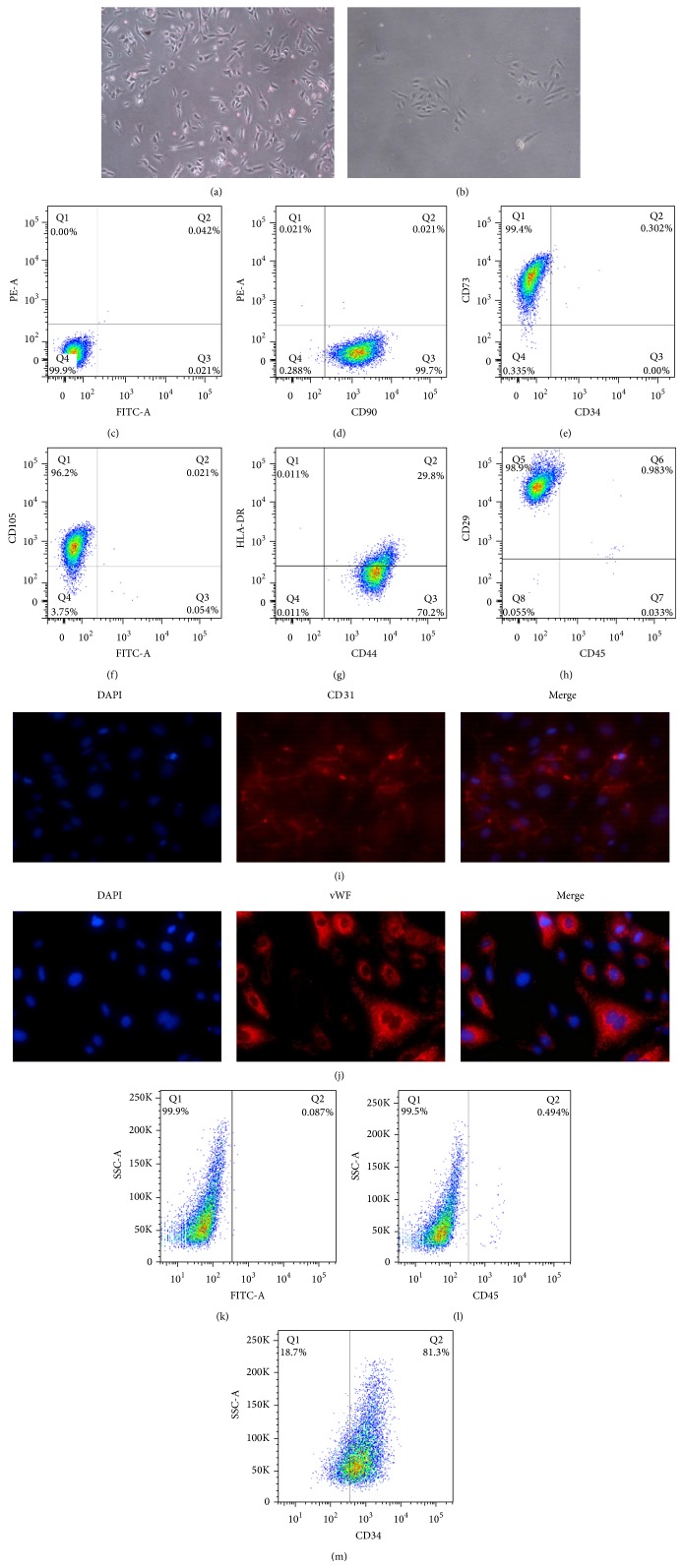
Identification of hAMSCs and HUVEC. Microscopic view (40^*^) of hAMSCs at P0 (a). Microscopic view (40^*^) of HUVECs at P0 (b). Surface marker analysis of freshly isolated hAMSCs by flow cytometry (c–h). The hAMSCs were positive for CD29, CD44, CD73, CD90, and CD105, while negative for CD45, CD34, and HLA-DR.  Figure 1(c) acted as negative control for Figures 1(d), 1(e), 1(f), 1(g), and 1(h). The isolated HUVECs expressed CD31 on membrane surface and vWF in cytoplasm (i and j), while they were positive for CD34 and negative for CD45 (m and l).  Figure 1(k) acted as negative control for Figures 1(l) and 1(m).

After 2 days in culture, hAMSCs displayed fibroblastic morphology (Figure 1(a)), which was similar to that observed in BMMSCs, and HUVEC displayed cobblestone-like morphology (Figure 1(b)). Surface markers of hAMSCs and HUVECs were evaluated by flow cytometry analysis at passage 2, respectively. It has been shown that hAMSCs were positive for MSC markers, such as CD29, CD44, CD73, CD90, and CD105, while negative for hematopoietic and vascular cell-related markers, such as CD45, CD34, and MHC class II antigen, such as HLA-DR (Figures 1(c)–1(h)). HUVECs were positive for CD34 and negative for CD45 (Figures 1(l) and 1(m)). Further identification of HUVECs was confirmed by immunofluorescence staining of vWF and CD31 (Figures 1(i) and 1(j)).

### 3.2. Histomorphometry of Implants* In Vivo*

**Figure 2:**
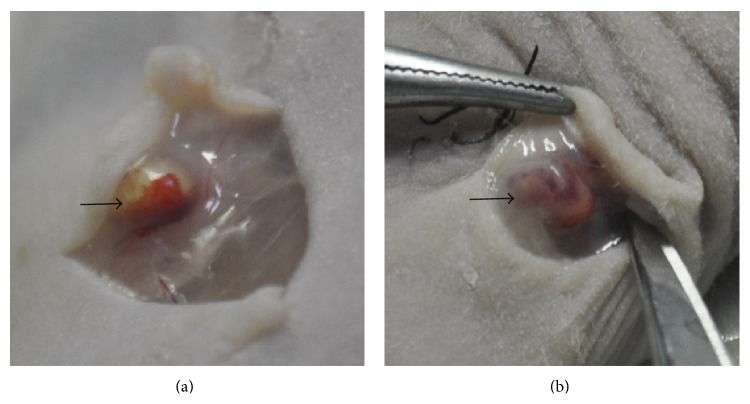
Explantation of* in vivo* implants on day 7 and day 14. (a) Harvesting on day 7. (b) Harvesting on day 14. Arrows show the vessel surrounding or penetrating inside the implants.

**Figure 3:**
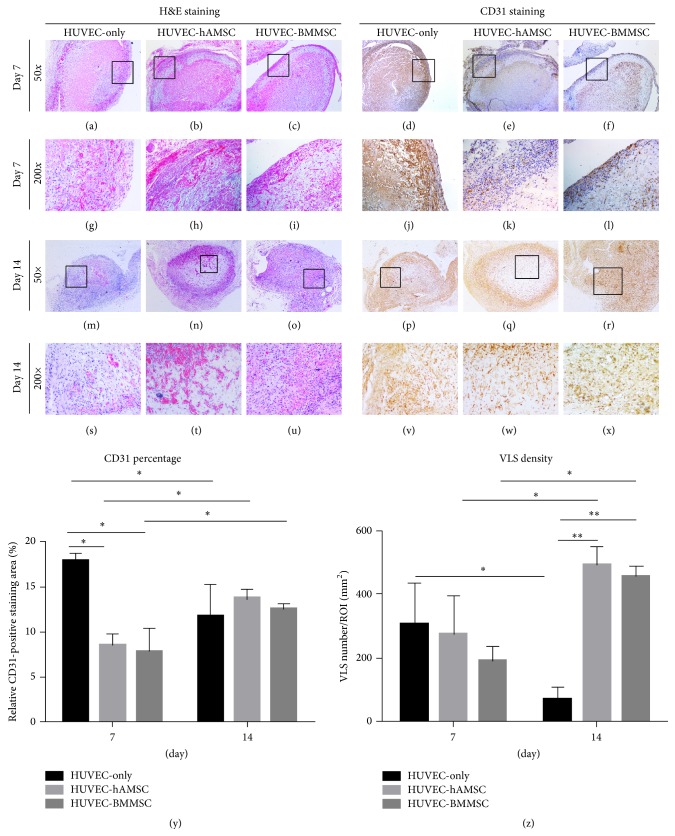
Histomorphometry of implants* in vivo*. H&E and CD31 staining illustrated varying blood vessel morphologies in implants in microscopic view (50^*^ and 200^*^). Stained sections from the day-7 implants contained vessel-like structures (VLS) in the outer layer. Vessel-like structures (VLS) can be found within stained sections from the day-14 implants. Quantification of the angiogenic capacity of HUVEC-only, HUVEC-hAMSC, and HUVEC-BMMSC is presented by CD31 percentage and VLS density (y and z).

The implants on the dorsal flank of the nude mice were collected at day 7 and day 14 (Figure 2). Images of the H&E-stained day-7 and day-14 implants showed differences on quantity, distribution, and morphology of the vessels formed in the various experimental groups. Stained sections from the day-7 implants contained vessel-like structures (VLS) in the outer layer, but these structures lacked in the inner one, and the implants contained obvious extravascular erythrocytes (Figures 3(g), 3(h), and 3(i)). Moreover, more vessel-like structures can be found within stained sections from the day-14 implants (Figures 3(w) and 3(x)). The vessel-like structures of HUVEC-only implants lacked consistent, circumscribed geometry, while the cells remained in the fibrin by day 14, and there were few capillary structures. By contrast, implants containing the MSCs (hAMSCs or BMMSCs) displayed clearly different tendency in images of the H&E-stained day-7 and day-14 implants. The samples in day-7 implants also contained many vessel-like structures in the outer layer and some atypical capillary structures, and many small capillaries with very well-defined lumens and circumscribed borders were investigated not only in outer layer but also in the center of the samples in day-14 implants. It was obvious that implants containing the MSCs had more capillary structures in the center compared with HUVEC-only implants. These capillaries were distributed throughout the entire implant to produce a vascularized implant containing both large and small blood vessels to effectively supply the tissue with oxygenated blood.

To verify the results from the H&E-stained sections, immunohistochemical staining for human CD31 was used to confirm the human origins of the neovascularization. In the implants of HUVECs-only group, there was a diffuse brown stain indicating an abundance of HUVECs and some lumen-like structures in the outer area of implants at days 7 and 14 (Figure 3). By contrast, the HUVEC-hAMSC and HUVEC-BMMSC implants contained many smaller, tightly sealed capillaries in the inner as well as outer layer, consistent with the observations from H&E staining. Quantification of the angiogenic capacity of three groups is presented in Figures 3(y) and 3(z). Both the comparison between the three groups and differences in angiogenesis over time (i.e., day 7 versus day 14) were evaluated. The increase of CD31-positive stained area was found in the comparison between the two types of cocultures at the two time points, but a significant decrease was found in HUVEC-only group. With regard to VLS density, VLS density of both HUVEC-hAMSC group and HUVEC-BMMSC group at day 14 was obviously higher than that at day 7. In addition, a significant decrease in VLS density was observed for HUVEC-only group between two time points.

### 3.3. Development of HUVEC Tube Formation under Serum-Free 3D Fibrin Matrices

**Figure 4:**
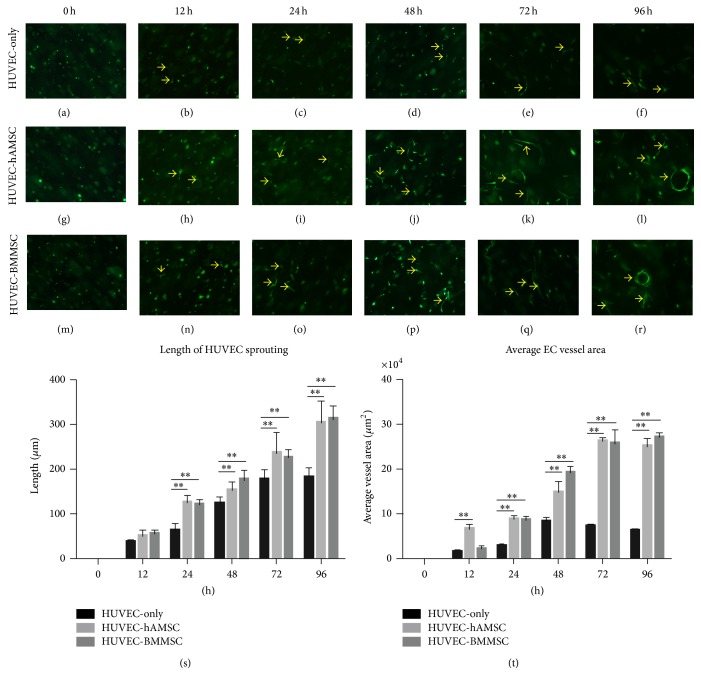
Development of HUVEC tube formation under serum-free 3D fibrin matrices. HUVECs were labeled with GFP and cocultured with MSCs (hAMSCs and BMMSCs). HUVEC-sprouting could be found as early as 12 h (indicated by arrows) and obviously at 48 h (indicated by arrows). Lumen-like structures rounded by HUVECs were indicated by arrows in (l) and (r). Microscopic view is 200^*^. The length of HUVEC-sprouting and average EC vessel area were showed in (s) and (t).

According to the results of experiment* in vivo*, we used a system [30] in which vascular tube morphogenesis and pericyte recruitment occurred in 3D fibrin matrices under serum-free defined conditions and using 96-well plates to observe the development of capillary tubes and the function of MSCs as pericyte. In this assay, HUVECs were labeled with GFP prior to 3D culture set-up and monocultured or cocultured with MSCs in 3D fibrin gels. Cultures were established at a 4 : 1 HUVEC : MSC ratio and were imaged using inverted fluorescence microscopy at six timepoints (i.e., 0 h, 12 h, 24 h, 48 h, 72 h, and 96 h) (Figure 4). HUVECs invaded extracellular matrix as early as 12 h and formed obvious sprouting by 48 h, but the processes of HUVEC tube formation and sprouting seemed to be limited without MSCs in 3D matrices. By contrast, extensive and increasing vascular networks were observed in both coculture conditions (HUVEC-hAMSC and HUVEC-BMMSC) from 24 h to 96 h. In the light sight that was same as the fluorescent sight, networks were more intensive in coculture conditions, while no network was investigated, and just ECs-sprouting in HUVEC-monoculture during the observation. Lumen-like structures rounded by HUVECs were detected at 96 h timepoint in two coculture systems (Figures 4(l) and 4(r)). To compare fluorescence sight with light sight, there were intensive networks comprised of MSCs surrounding the lumen-like structures. The phenotype might indicate that hAMSCs had equal capacity with BMMSCs to enhance HUVEC-sprouting and vascular networks forming, and MSCs had especially important meaning to stabilize the networks.

**Figure 5:**
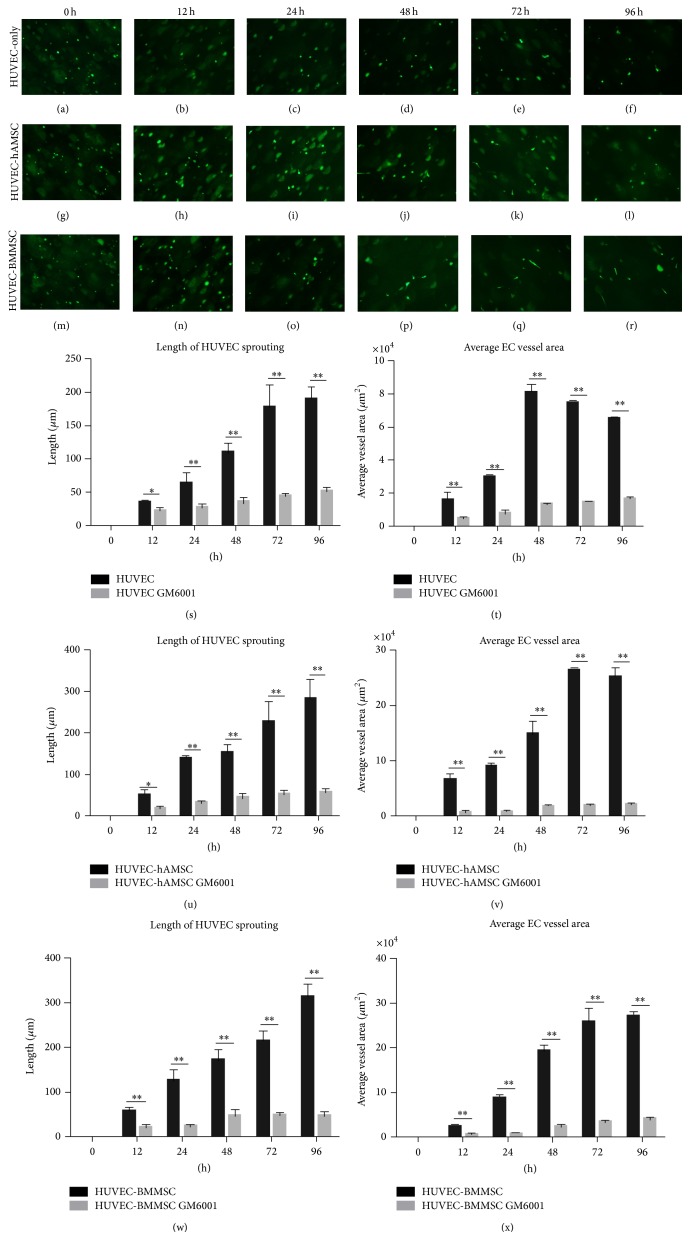
Inhibitory effect of MMPs in HUVEC tube formation under serum-free 3D fibrin matrices. HUVEC-sprouting had been inhibited significantly across all timepoints (a–r). Microscopic view is 200^*^. The length of HUVEC-sprouting and average EC vessel area were decreased remarkably (s–x). There was no significant difference among three groups.

Localized proteolytic activity is necessary to facilitate key steps of the angiogenic cascade [31, 32]. Matrix metalloproteinases (MMPs) serve a purpose in regulating capillary diameter and possibly in stabilizing the nascent vessels [17]. These proteolytic mechanisms are involved in fibroblast-mediated angiogenesis and in BMMSC-mediated angiogenesis [15]. To test the proteolytic mechanisms related to hAMSC-mediated angiogenesis, 3D culture system was treated with the broad-spectrum MMP inhibitor GM6001. As shown in Figure 5, broad scale inhibition of MMPs had significant effect on HUVECs tube formation and sprouting. In HUVEC-only with GM6001, there was no obvious HUVECs-sprouting at several time points. HUVECs-sprouting of HUVEC-MSC coculture with GM6001 increased slightly, but still significantly less than the coculture without GM6001. Interestingly, although the processes of HUVEC tube formation and sprouting were inhibited, the networks formed by MSCs still existed. These phenotypes might suggest the proteolytic mechanism related to hAMSC-mediated angiogenesis was similar to that related to BMMSC, and, on the other hand, stabilization of vascular networks supported by MSCs might be to form MSC networks.

### 3.4. MMPs Expression of 3D Cell Clusters

**Figure 6:**
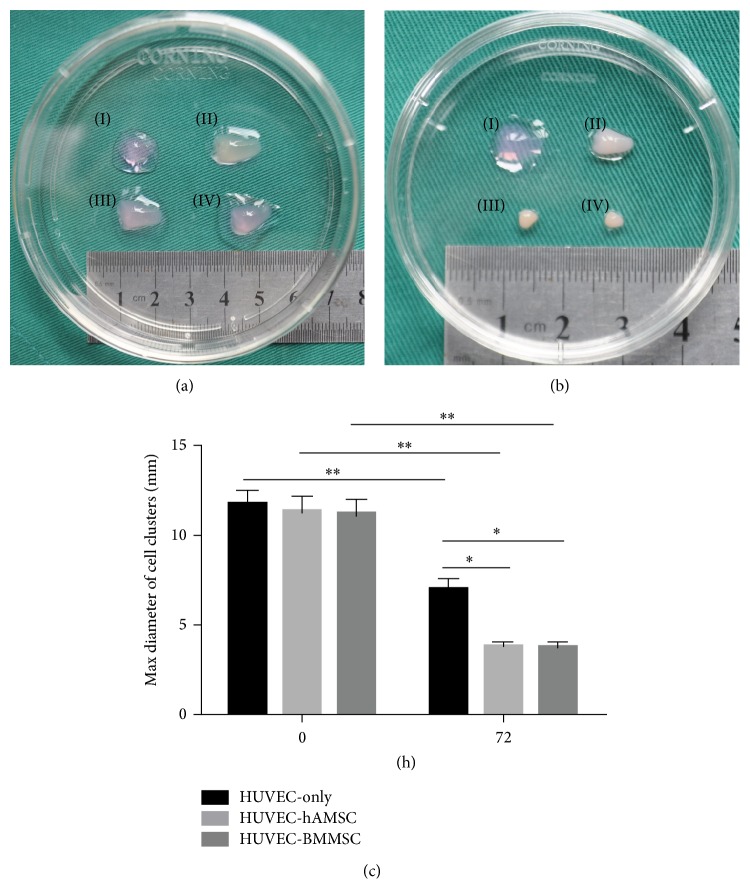
3D cluster* in vitro*. (a) Prepared 3D clusters. Fibrin gel without cells (I), 3D cluster of HUVEC-only (II), 3D cluster of HUVEC-hAMSC (III), and 3D cluster of HUVEC-BMMSC (IV); (b) 3D clusters after culturing 3 days in *α*-MEM. Fibrin gel without cells (I), 3D cluster of HUVEC-only (II), 3D cluster of HUVEC-hAMSC (III), and 3D cluster of HUVEC-BMMSC (IV). (c) Max diameter of cell clusters. This experiment was repeated three times and a representative sample was shown.

**Figure 7:**
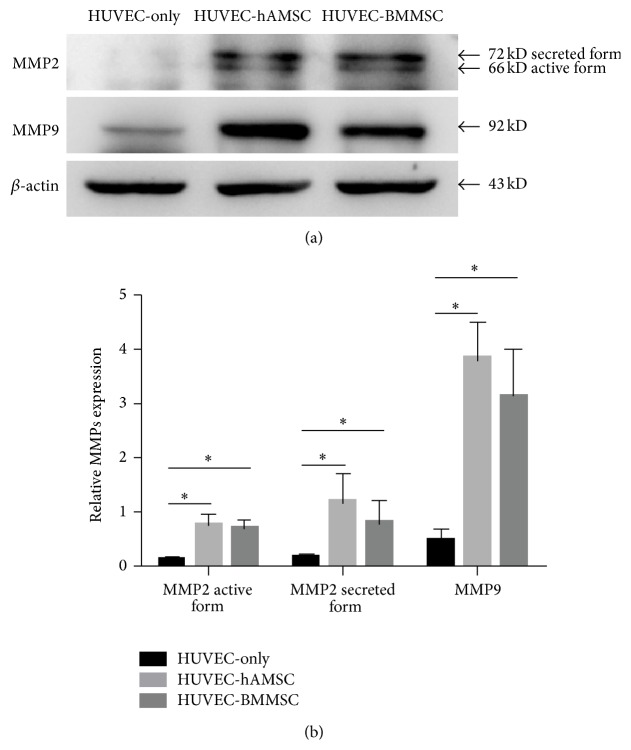
MMP2, MMP9 expression in 3D clusters. The MMP2, MMP9 and *β*-actin (internal control) expression in HUVEC-only clusters, HUVEC-hAMSC clusters, and HUVEC-BMMSC clusters were probed via Western blotting (day 3). This experiment was repeated three times and a representative sample was shown (a). The relative expression of MMPs was analyzed (b).

As shown in Figure 4, ECs cultured in the absence of MSCs do not form capillary-like structures in fibrin gels, but intensive networks and lumen-like structures were observed in both HUVEC-hAMSC and HUVEC-BMMSC cocultures. With regard to BMMSC-mediated proteolytic mechanisms, MMPs play a key role in resolving extracellular matrix and promoting HUVEC-sprouting. To study the function of MSCs in 3D coculture condition, we observed the 3D clusters of 3 groups (HUVEC-only, HUVEC-hAMSC, and HUVEC-BMMSCs)* in vitro* after culturing for three days in *α*-MEM. At day 3, the samples of the gels shrunk in different degree, but the gels containing MSCs shrunk more significantly as shown in Figure 6. There was no change in gels without cells by 3-day culture. On the other hand, the results of Western blotting showed that the expression of active-MMP2 in HUVEC-hAMSC and HUVEC-BMMSC was about 4-fold more than that in HUVEC-only, while the expression of secreted MMP2 in HUVEC-hAMSC and HUVEC-BMMSC was about 5-fold more than that in HUVEC-only. The similar trend was shown in expression of MMP9 among three groups (Figure 7).

## 4. Discussion

In this study, we investigated the angiogenic capacity of hAMSC/HUVEC coculture and the function of hAMSC on proteolytic mechanisms both* in vivo *and* in vitro*. Firstly, we demonstrated that hAMSC/HUVEC coculture shared equal angiogenic capacity with BMMSC/HUVEC coculture* in vivo*. Secondly, it was similar to BMMSCs that hAMSCs could enhance the processes of HUVEC tube formation and sprouting* in vitro*. Thirdly, MMPs were found in hAMSC-mediated proteolytic mechanism shared with BMMSC-mediated proteolytic processes. Fourthly, MMPs expression levels of hAMSCs could be elevated significantly in 3D culture condition.

Mesenchymal stem cells were identified in human postnatal bone marrow (BM) and later in peripheral blood, periosteum, muscle, adipose tissue, and connective tissue of human adults [33–37]. Traditional source of MSCs for clinical investigations is BM. Extensive studies of BM-derived MSCs (BMMSCs) have proven their multipotent differentiation potential and powerful immunosuppressive qualities [38]. However, the collection of BM is associated with invasive procedures involving significant discomfort to the patient. Moreover, it results in a relatively low amount of MSCs (approximately 0.001–0.01% of all isolated nuclear cells) in adult human BM, and the number of cells decreases with donor's age [39]. Besides, ethics problems are also the important aspect of restricting its application. Because there are no ethical problems involved, using hAMSCs as an allogeneic stem cell source should be highly beneficial. They can be collected easily, have multipotential capacity, and express only low levels of the major histocompatibility complex (MHC) class I antigens and be negative for MHC class II antigens on their surface [40]. The MHC expression of BMMSCs can be induced by treating with interferon-*γ*, but that of hAMSCs is much lower than BMMSCs [41, 42]. For several years, BMMSCs have been considered as immune privileged cells, unable to induce alloreactivity in humans. However, more recently it has been demonstrated that donor-derived MSCs are immunogenic in an allogeneic host and stimulate graft rejection in a murine model of submyeloablative allogeneic BM transplantation [43]. Immunogenicity of hAMSCs seems to be better than that of BMMSCs. Like BMMSCs, hAMSCs can adhere and proliferate on tissue culture plastic, present fibroblast-like appearance, form clonal colonies, and express the typical range of BMMSC associated cell surface markers, such as CD29, CD44, CD49e, CD73, CD90, and CD105, while being negative for hematopoietic and vascular cell-related markers, such as CD45, CD34 [44]. Regarding HUVEC, it has been well-known that von Willebrand Factor (vWF), platelet-endothelial adhesion molecule-1 (PECAM-1; CD31), and CD34 are specific markers for endothelial cells [45, 46] and negative for human CD45, a tyrosine phosphatase also known as the leukocyte common antigen (LCA). The CD45 molecule is required for T and B cell activation and is expressed in at least five isoforms depending on the differentiation status of the cell. In the present study, identification of isolated hAMSCs and HUVECs was consistent with the conclusion published previously.

A number of studies have demonstrated that the direct contact and communication between ECs and pericyte-like cells are essential for vascularization [47, 48]. AdMSCs can secrete a broad range of paracrine factors that are known to be angiogenic, similar to BMMSCs [49]. Human umbilical vein endothelial cells (HUVECs) are the most commonly used type of human endothelial cells (ECs) for both* in vitro *and* in vivo *studies for bone regenerative medicine [50]. Both BMMSCs and AdMSCs can play an active role in the formation, stabilization, and maturation of newly formed VLS [51, 52] and share equal angiogenic capacity both* in vitro* and* in vivo*, and vessels from donor origin can anastomose with the host vasculature within seven days of implantation [29]. Thus, it was hypothesized that hAMSCs also had similar function like BMMSCs and AdMSCs on neovascularization. In this study, cells were cultured in direct contact according to HUVECs : MSCs as 1 : 1 ratio* in vivo* [9, 29]; the angiogenic capacities of HUVEC-only, HUVECs-hAMSCs, and HUVEC-BMMSCs were examined at two time points (i.e., day 7 and day 14) by H&E and hCD31 staining. The implants containing HUVECs alone appeared to yield greater numbers of vessel-like structures initially, but these vessels were unstable in the absence of a codelivered stromal cell. One of possible reasons was that vessels cannot be formed by HUVEC alone; pericytes provided the essential support for HUVECs to construct and stabilize newly formed vessels. Moreover, many lumen-like structures in the interior of implants of both HUVECs-hAMSCs groups and HUVEC-BMMSCs groups might reflect the function of hAMSCs on angiogenic process* in vivo* was similar to BMMSCs that MSCs as pericytes played an auxiliary role on helping HUVECs to degrade extracellular matrix and form mature capillaries.

To further detect the mechanism of hAMSC on angiogenic process, HUVEC-monoculture and HUVEC-MSCs cocultures were set up* in vitro*. hAMSCs cultured in endothelial induction conditions showed upregulation of antiangiogenic and concomitant downregulation of proangiogenic genes and proteins to protect themselves against differentiating into mature endothelial cells; however, the conditioned media of induced hAMSC had a positive effect on endothelial cells as shown by enhanced viability and stabilized network formation [28]. These results are consistent with studies showing that BMMSCs promote angiogenesis and support blood vessel formation [51, 53, 54]. In the present study, we seeded cells in the fibrin gels prepared with endothelial cell growth medium, which contained a variety of endothelial cell growth factors, and showed that hAMSC and BMMSC could constitute intensive and stable network to have a positive effect on vascular network in endothelial induction condition. On the other hand, it has been well-established that MMPs or/and the PA/plasmin axis play important roles in EC invasion during capillary morphogenesis; however, different pericyte types influence the proteolytic ways via different mechanisms. BMMSC-mediated proteolytic mechanisms had been demonstrated to be solely related to MMPs [15]. In our present work, we utilized coculture model treated or not treated with GM6001 to imitate early phrase of angiogenesis* in vitro* to demonstrate that both hAMSCs and BMMSCs stimulate capillary morphogenesis within 3D fibrin ECMs and seem to do via same proteolytic mechanism. The similar behavior of HUVEC-hAMSC and HUVEC-BMMSCs cocultures treated or not treated with GM6001 in fluorescence field and expression of MMP2 and MMP9 in coculture gels might suggest that hAMSC-mediated angiogenesis was related to MMPs, just like BMMSC-mediated angiogenesis mechanisms.

With the hope of future clinical application, there are many researches concentrating on stem cells from different sources. Stem cells are undifferentiated cells that are found in the embryonic, fetal, and adult stages of life and have become a novel hope in cell-based therapy [55, 56]. Stem cells from amniotic fluid (AF) and amniotic membrane (AM) can be used in clinical therapeutic applications without ethical limitations in the future [57]. This study demonstrated that hAMSC and BMSCs as pericytes sharing equal ability of enhancing angiogenesis via MMPs that impact the functional qualities of the capillary networks both* in vitro* and* in vivo* and stabilized new capillaries branch during the early stage. On the other hand, hAMSCs can secrete cytokines engaged in angiogenesis, osteogenesis, and anti-inflammation, such as VEGF [40], TGF-*β* [58], PGE2 [58], IL-1 receptor agonist [59], and IL-10 [59]. Moreover, bone morphogenetic proteins (BMPs), the members of TGF-*β* superfamily, are highly conserved signaling molecules that have been well-established for the function in the patterning and morphogenesis of many organs including bone regeneration [60]. It is thus conceivable that the positive effect of hAMSCs in angiogenesis should have a profound meaning in bone tissue regeneration and other clinic applications, even though many challenges should be conquered.

In conclusion, hAMSCs might be as valuable as BMMSC in a variety of cell-therapeutic and tissue engineering applications since they could promote the survival of endothelial cells and the stabilization of vascular networks. In future studies, the more effect and significance of hAMSC on tissue engineering should be investigated more comprehensively and in-depth.

## References

[1] Petite H., Viateau V., Bensaïd W. (2000). Tissue-engineered bone regeneration. *Nature Biotechnology*.

[2] Santos M. I., Reis R. L. (2010). Vascularization in bone tissue engineering: physiology, current strategies, major hurdles and future challenges. *Macromolecular Bioscience*.

[3] Novosel E. C., Kleinhans C., Kluger P. J. (2011). Vascularization is the key challenge in tissue engineering. *Advanced Drug Delivery Reviews*.

[4] Lovett M., Lee K., Edwards A., Kaplan D. L. (2009). Vascularization strategies for tissue engineering. *Tissue Engineering, Part B: Reviews*.

[5] Ferrara N., Alitalo K. (1999). Clinical applications of angiogenic growth factors and their inhibitors. *Nature Medicine*.

[6] Sun Q., Chen R. R., Shen Y., Mooney D. J., Rajagopalan S., Grossman P. M. (2005). Sustained vascular endothelial growth factor delivery enhances angiogenesis and perfusion in ischemic hind limb. *Pharmaceutical Research*.

[7] Zisch A. H., Lutolf M. P., Ehrbar M. (2003). Cell-demanded release of VEGF from synthetic, biointeractive cell ingrowth matrices for vascularized tissue growth. *The FASEB Journal*.

[8] Sun Q., Silva E. A., Wang A. (2010). Sustained release of multiple growth factors from injectable polymeric system as a novel therapeutic approach towards angiogenesis. *Pharmaceutical Research*.

[9] Grainger S. J., Carrion B., Ceccarelli J., Putnam A. J. (2013). Stromal cell identity influences the in vivo functionality of engineered capillary networks formed by co-delivery of endothelial cells and stromal cells. *Tissue Engineering Part A*.

[10] Rehman J., Traktuev D., Li J. (2004). Secretion of angiogenic and antiapoptotic factors by human adipose stromal cells. *Circulation*.

[11] Kinnaird T., Burnett E. S., Shou M. (2004). Local delivery of marrow-derived stromal cells augments collateral perfusion through paracrine mechanisms. *Circulation*.

[12] Pesce M., Orlandi A., Iachininoto M. G. (2003). Myoendothelial differentiation of human umbilical cord blood-derived stem cells in ischemic limb tissues. *Circulation Research*.

[13] Ren L., Ma D., Liu B. (2014). Preparation of three-dimensional vascularized MSC cell sheet constructs for tissue regeneration. *BioMed Research International*.

[14] Ghajar C. M., Blevins K. S., Hughes C. C. W., George S. C., Putnam A. J. (2006). Mesenchymal stem cells enhance angiogenesis in mechanically viable prevascularized tissues via early matrix metalloproteinase upregulation. *Tissue Engineering*.

[15] Ghajar C. M., Kachgal S., Kniazeva E. (2010). Mesenchymal cells stimulate capillary morphogenesis via distinct proteolytic mechanisms. *Experimental Cell Research*.

[16] Au P., Tam J., Fukumura D., Jain R. K. (2008). Bone marrow derived mesenchymal stem cells facilitate engineering of long-lasting functional vasculature. *Blood*.

[17] Kachgal S., Putnam A. J. (2011). Mesenchymal stem cells from adipose and bone marrow promote angiogenesis via distinct cytokine and protease expression mechanisms. *Angiogenesis*.

[18] Merfeld-Clauss S., Gollahalli N., March K. L., Traktuev D. O. (2010). Adipose tissue progenitor cells directly interact with endothelial cells to induce vascular network formation. *Tissue Engineering—Part A*.

[19] Chen X., Aledia A. S., Popson S. A., Him L., Hughes C. C. W., George S. C. (2010). Rapid anastomosis of endothelial progenitor cell-derived vessels with host vasculature is promoted by a high density of cotransplanted fibroblasts. *Tissue Engineering—Part A*.

[20] Shepherd B. R., Jay S. M., Saltzman W. M., Tellides G., Pober J. S. (2009). Human aortic smooth muscle cells promote arteriole formation by coengrafted endothelial cells. *Tissue Engineering Part A*.

[21] Bergers G., Song S. (2005). The role of pericytes in blood-vessel formation and maintenance. *Neuro-Oncology*.

[22] Soncini M., Vertua E., Gibelli L. (2007). Isolation and characterization of mesenchymal cells from human fetal membranes. *Journal of Tissue Engineering and Regenerative Medicine*.

[23] Ilancheran S., Moodley Y., Manuelpillai U. (2009). Human fetal membranes: a source of stem cells for tissue regeneration and repair?. *Placenta*.

[24] de Girolamo L., Lucarelli E., Alessandri G. (2013). Mesenchymal stem/stromal cells: a new “cells as drugs” paradigm. efficacy and critical aspects in cell therapy. *Current Pharmaceutical Design*.

[25] Alviano F., Fossati V., Marchionni C. (2007). Term amniotic membrane is a high throughput source for multipotent mesenchymal stem cells with the ability to differentiate into endothelial cells in vitro. *BMC Developmental Biology*.

[26] Kim S.-W., Zhang H.-Z., Kim C. E., An H. S., Kim J.-M., Kim M. H. (2012). Amniotic mesenchymal stem cells have robust angiogenic properties and are effective in treating hindlimb ischaemia. *Cardiovascular Research*.

[27] Li Y., Guo L., Ahn H. S., Kim M. H., Kim S.-W. (2014). Amniotic mesenchymal stem cells display neurovascular tropism and aid in the recovery of injured peripheral nerves. *Journal of Cellular and Molecular Medicine*.

[28] König J., Huppertz B., Desoye G. (2012). Amnion-derived mesenchymal stromal cells show angiogenic properties but resist differentiation into mature endothelial cells. *Stem Cells and Development*.

[29] Ma J., Yang F., Both S. K. (2014). In vitro and in vivo angiogenic capacity of BM-MSCs/HUVECs and AT-MSCs/HUVECs cocultures. *Biofabrication*.

[30] Smith A. O., Bowers S. L. K., Stratman A. N., Davis G. E. (2013). Hematopoietic stem cell cytokines and fibroblast growth factor-2 stimulate human endothelial cell-pericyte tube co-assembly in 3D fibrin matrices under serum-free defined conditions. *PLoS ONE*.

[31] Hiraoka N., Allen E., Apel I. J., Gyetko M. R., Weiss S. J. (1998). Matrix metalloproteinases regulate neovascularization by acting as pericellular fibrinolysins. *Cell*.

[32] Yana I., Sagara H., Takaki S. (2007). Crosstalk between neovessels and mural cells directs the site-specific expression of MT1-MMP to endothelial tip cells. *Journal of Cell Science*.

[33] Pittenger M. F., Mackay A. M., Beck S. C. (1999). Multilineage potential of adult human mesenchymal stem cells. *Science*.

[34] Zuk P. A., Zhu M., Ashjian P. (2002). Human adipose tissue is a source of multipotent stem cells. *Molecular Biology of the Cell*.

[35] Zvaifler N. J., Marinova-Mutafchieva L., Adams G. (2000). Mesenchymal precursor cells in the blood of normal individuals. *Arthritis Research*.

[36] Nakahara H., Bruder S. P., Haynesworth S. E. (1990). Bone and cartilage formation in diffusion chambers by subcultured cells derived from the periosteum. *Bone*.

[37] Young H. E., Steele T. A., Bray R. A. (2001). Human reserve pluripotent mesenchymal stem cells are present in the connective tissues of skeletal muscle and dermis derived from fetal, adult, and geriatric donors. *Anatomical Record*.

[38] Harichandan A., Bühring H.-J. (2011). Prospective isolation of human MSC. *Best Practice and Research: Clinical Haematology*.

[39] Isern J., Méndez-Ferrer S. (2011). Stem cell interactions in a bone marrow niche. *Current Osteoporosis Reports*.

[40] Kmiecik G., Niklińska W., Kuć P. (2013). Fetal membranes as a source of stem cells. *Advances in Medical Sciences*.

[41] le Blanc K., Tammik C., Rosendahl K., Zetterberg E., Ringdén O. (2003). HLA expression and immunologic properties of differentiated and undifferentiated mesenchymal stem cells. *Experimental Hematology*.

[42] Götherström C., Ringdén O., Tammik C., Zetterberg E., Westgren M., Le Blanc K. (2004). Immunologic properties of human fetal mesenchymal stem cells. *American Journal of Obstetrics & Gynecology*.

[43] Nauta A. J., Westerhuis G., Kruisselbrink A. B., Lurvink E. G. A., Willemze R., Fibbe W. E. (2006). Donor-derived mesenchymal stem cells are immunogenic in an allogeneic host and stimulate donor graft rejection in a nonmyeloablative setting. *Blood*.

[44] Zhang Y., Li C., Jiang X. (2004). Human placenta-derived mesenchymal progenitor cells support culture expansion of long-term culture-initiating cells from cord blood CD34^+^ cells. *Experimental Hematology*.

[45] Albelda S. M., Muller W. A., Buck C. A., Newman P. J. (1991). Molecular and cellular properties of PECAM-1 (endoCAM/CD31): a novel vascular cell-cell adhesion molecule. *The Journal of Cell Biology*.

[46] Girma J.-P., Meyer D., Verweij C. L., Pannekoek H., Sixma J. J. (1987). Structure-function relationship of human von Willebrand factor. *Blood*.

[47] Cai X., Lin Y., Friedrich C. C. (2009). Bone marrow derived pluripotent cells are pericytes which contribute to vascularization. *Stem Cell Reviews*.

[48] Rao R. R., Peterson A. W., Ceccarelli J., Putnam A. J., Stegemann J. P. (2012). Matrix composition regulates three-dimensional network formation by endothelial cells and mesenchymal stem cells in collagen/fibrin materials. *Angiogenesis*.

[49] Hsiao S. T.-F., Asgari A., Lokmic Z. (2012). Comparative analysis of paracrine factor expression in human adult mesenchymal stem cells derived from bone marrow, adipose, and dermal tissue. *Stem Cells and Development*.

[50] Alard J.-E., Dueymes M., Mageed R. A., Saraux A., Youinou P., Jamin C. (2009). Mitochondrial heat shock protein (HSP) 70 synergizes with HSP60 in transducing endothelial cell apoptosis induced by anti-HSP60 autoantibody. *The FASEB Journal*.

[51] Verseijden F., Posthumus-van Sluijs S. J., Pavljasevic P., Hofer S. O. P., van Osch G. J. V. M., Farrell E. (2010). Adult human bone marrow-and adipose tissue-derived stromal cells support the formation of prevascular-like structures from endothelial cells in vitro. *Tissue Engineering—Part A*.

[52] Sacchetti B., Funari A., Michienzi S. (2007). Self-renewing osteoprogenitors in bone marrow sinusoids can organize a hematopoietic microenvironment. *Cell*.

[53] Kinnaird T., Stabile E., Burnett M. S. (2004). Marrow-derived stromal cells express genes encoding a broad spectrum of arteriogenic cytokines and promote in vitro and in vivo arteriogenesis through paracrine mechanisms. *Circulation Research*.

[54] Gruber R., Kandler B., Holzmann P. (2005). Bone marrow stromal cells can provide a local environment that favors migration and formation of tubular structures of endothelial cells. *Tissue Engineering*.

[55] Volarevic V., Bojic S., Nurkovic J. (2014). Stem cells as new agents for the treatment of infertility: current and future perspectives and challenges. *BioMed Research International*.

[56] Jin M., Xie Y., Li Q., Chen X. (2014). Stem cell-based cell therapy for glomerulonephritis. *BioMed Research International*.

[57] Roubelakis M. G., Trohatou O., Anagnou N. P. (2012). Amniotic fluid and amniotic membrane stem cells: marker discovery. *Stem Cells International*.

[58] Rossi D., Pianta S., Magatti M., Sedlmayr P., Parolini O. (2012). Characterization of the conditioned medium from amniotic membrane cells: prostaglandins as key effectors of its immunomodulatory activity. *PLoS ONE*.

[59] Silini A., Parolini O., Huppertz B., Lang I. (2013). Soluble factors of amnion-derived cells in treatment of inflammatory and fibrotic pathologies. *Current Stem Cell Research and Therapy*.

[60] Hussain A., Takahashi K., Sonobe J., Tabata Y., Bessho K. (2014). Bone regeneration of rat calvarial defect by magnesium calcium phosphate gelatin scaffolds with or without bone morphogenetic protein-2. *Journal of Maxillofacial and Oral Surgery*.

